# Reviewer acknowledgement 2016

**DOI:** 10.1186/s13148-016-0187-4

**Published:** 2016-02-17

**Authors:** Ulrich Mahlknecht

**Affiliations:** Department for Internal Medicine- Hematology/Oncology, St Lukas Clinic, Solingen, Germany; Department of Internal Medicine, Division of Immunotherapy and Gene Therapy, Saarland University Medical Center, Homburg, Germany

## Abstract

The editors of *Clinical Epigenetics* would like to thank all our reviewers who have contributed to the journal in Volume 7 (2015).

**Hany Abdel-Hafiz**

United States of America

**Charbel Abi Khalil**

United States of America

**Nita Ahuja**

United States of America

**Neil Alexis**

United States of America

**Elizabeth Algar**

Australia

**Markus Almen**

Sweden

**Kristian Almstrup**

Denmark

**Lucia Altucci**

Italy

**Concetta Ambrosino**

Italy

**Ole Ammerpohl**

Germany

**Cindy Anderson**

United States of America

**Takahiro Arima**

Japan

**Paola Arimondo**

France

**Hideo Baba**

Japan

**Francesc Balaguer**

Spain

**Alfonso Baldi**

Italy

**Ajay Bansal**

United States of America

**Ludovic Barault**

Italy

**Romain Barrès**

Denmark

**Marisa Bartolomei**

United States of America

**Subit Barua**

United States of America

**Christopher Bell**

United Kingdom

**Jordana Bell**

United Kinhdom

**Rosaria Benedetti**

Italy

**Mark Bettington**

Australia

**András Bikov**

Hungary

**Giovanni Blandino**

Italy

**Christoph Bock**

Germany

**Linda Booij**

Canada

**Yvonne Böttcher**

Germany

**Sophie Bouchat**

Belgium

**Pierre Bougneres**

France

**Mariana Brait**

United States of America

**Elizabeth Braithwaite**

United Kingdom

**Lutz Breitling**

Germany

**Karin Broberg**

Sweden

**Hannah Brown**

Australia

**Robert Brown**

United Kingdom

**Michael Buckland**

Australia

**Stefan Burdach**

Germany

**Heather Burris**

United States of America

**Christian Busch**

Germany

**Paul Cairns**

United States of America

**Daniele Calistri**

Italy

**Monica Cappetta**

Uruguay

**Michele Caraglia**

Italy

**Jason Carlyon**

United States of America

**André Carvalho**

United States of America

**Biman Chakraborty**

United Kingdom

**Michael Chan**

Taiwan

**Brian Chen**

United States of America

**Xiangmei Chen**

China

**YW Chen**

United States of America

**Travers Ching**

United States of America

**Brock Christensen**

United States of America

**Maximilian Christopeit**

Germany

**Steven Clarke**

United States of America

**Tine Dalsgaard Clausen**

Denmark

**Sarah Cohen-Woods**

Austria

**William Coleman**

United States of America

**Pablo Conesa-Zamora**

Spain

**Elizabeth Conradt**

United States of America

**Mariarosaria Conte**

Italy

**Fabio Coppede**

Italy

**Sabrina Corbetta**

Italy

**Rene Cortese**

United States of America

**Ivan Costa**

Germany

**Bruno Costa**

Portugal

**Christos Coutifaris**

United States of America

**Jeffrey Craig**

Australia

**Marion Cremer**

Germany

**Teresa Cunha-Oliveira**

Portugal

**Edward Curry**

United Kingdom

**Ingrid Dahlman**

Sweden

**Roderick Dashwood**

United States of America

**Dario De Biase**

Italy

**Simone de Jong**

United Kingdom

**Vanessa de Mello Laaksonen**

Finland

**Susanne de Rooij**

Netherlands

**Lissette Delgado Cruzata**

United States of America

**Floriana Della Ragione**

Italy

**Emma Dempster**

United Kingdom

**Christine Disteche**

United States of America

**Hester Doyle**

United States of America

**Zhenzong Du**

China

**Christophe Dupont**

France

**Gerda Egger**

Austria

**Thomas Eggermann**

Germany

**Rachel Eiges**

Italy

**Mattias Ekstedt**

Sweden

**Magdeldin Elgizouli**

Germany

**Lena Eliasson**

Sweden

**Elliot Epner**

United States of America

**Todd Everson**

United States of America

**Mary Jo Fackler**

United States of America

**Sanja A Farkas**

Sweden

**Robert Feil**

France

**Janine Felix**

Netherlands

**Weiwei Feng**

China

**Leonides Fernandez**

Spain

**Eduardo Fernandez-Rebollo**

Germany

**James Flanagan**

United Kingdom

**Michael Fleischhacker**

Germany

**Kirsty Flower**

United Kingdom

**Roger Foo**

United Kingdom

**Karen Forbes**

United Kingdom

**Delphine Fradin**

France

**Gianluigi Franci**

Italy

**Diha Freije**

United States of America

**Simonetta Friso**

Italy

**Mattia Frontini**

United Kingdom

**Rebecca Fry**

United States of America

**Ting Fu**

United States of America

**Tohru Fujiwara**

Japan

**Atsushi Fukuda**

Japan

**Tamas Fulop**

Canada

**Jochen Gaedcke**

Germany

**Thilo Gambichler**

Germany

**Yuzhen Gao**

China

**Regina Garcia**

Spain

**Lana Garmire**

United States of America

**Francine Garrett-Bakelman**

United States of America

**Niklas Gebauer**

Germany

**Maurizio Genuardi**

Italy

**Philippe Georgel**

United States of America

**Clarissa Gerhauser**

Germany

**Ugur Gezer**

Turkey

**Elisa Giovannetti**

Italy

**Marcus Gomes**

Brazil

**Zhiyun Gong**

China

**Maria Gonzalez Cao**

Spain

**Michael Gordon**

United Kingdom

**Steven Gray**

Ireland

**Benjamin Green**

United States of America

**Joerg Gromoll**

Germany

**Elin Grundberg**

Canada

**Rafael Guerrero-Preston**

United States of America

**Sonia Guil**

Spain

**Shicheng Guo**

United States of America

**Jan Haas**

Germany

**Michael Haffner**

United States of America

**Paul Haggarty**

United Kingdom

**John Halsall**

United Kingdom

**Toshio Hamatani**

Japan

**Qin Han**

China

**Eilis Hannon**

United Kingdom

**Sara Harlid**

United States of America

**Alexander Haslberger**

Austria

**Naoko Hattori**

Japan

**Shunmin He**

China

**Christopher Heeschen**

United Kingdom

**Rui Henrique**

Portugal

**Georges Herbein**

France

**Eric Hervouet**

France

**Kinjal Hew**

United States of America

**Holger Heyn**

Spain

**Toshinori Hinoue**

United States of America

**Hitoshi Hiura**

Japan

**Marie-France Hivert**

Canada

**Trang Hoang**

Canada

**Stefan Holdenrieder**

Denmark

**Nina Holland**

United States of America

**Shin-ichi Horike**

JAPAN

**Steve Horvath**

United States of America

**J-Fan Hu**

United States of America

**Yuhui Huang**

United States of America

**T Huang**

China

**Paula Hyland**

United Kingdom

**Vito Iacobazzi**

Italy

**Takuya Imamura**

Japan

**Miho Ishida**

United Kingdom

**Khaled Ismail**

United Kingdom

**Benjamin Jackson**

United Kingdom

**Bram Janssen**

Belgium

**Magdalene Jawahar**

Australia

**Paul Jedlicka**

United States of America

**Jaroslav Jelinek**

United States of America

**Albert Jeltsch**

Germany

**Carmen Jeronimo**

Portugal

**Victor Jin**

United States of America

**Ian Johnson**

United Kingdom

**Ricky Johnstone**

Australia

**Takako I Jones**

United States of America

**Peter Jones**

United States of America

**Jihoon Joo**

Australia

**Kerstin Junker**

Germany

**Jennifer Kalish**

United States of America

**Zachary Kaminsky**

United States of America

**Gyeong Hoon Kang**

South Korea

**Maya Kappil**

United States of America

**Pawel Karpinski**

Poland

**Jyotdeep Kaur**

India

**Richard Kelly**

United Kingdom

**Karl Kelsey**

United States of America

**Amy Kenter**

United States of America

**Joomyeong Kim**

United States of America

**Young-Joon Kim**

South Korea

**M Kimura**

Japan

**Amanda Kirkham**

United Kingdom

**Devin Koestler**

United States of America

**Dieuwertje Kok**

Netherlands

**Klaas Kok**

Netherlands

**Yutaka Kondo**

Japan

**Gabor L Kovacs**

Hungary

**Renu Kowluru**

United States of America

**Theo F. J. Kraus**

Germany

**Smriti Krishna**

Australia

**Xiong Kuangnan**

United States of America

**Agné Kulyté**

Sweden

**Amit Kumar**

France

**Robert Kumsta**

Germany

**Alina Kurylowicz**

Poland

**Matthew A. Kutny**

United States of America

**Bernard Kwabi-Addo**

United States of America

**Viviane Labrie**

Canada

**Hung-Cheng Lai**

Taiwan

**Rosa Lapalombella**

United States of America

**Janine LaSalle**

United States of America

**Martin Lauss**

Sweden

**Barbara Leggett**

Australia

**Ulrich Lehmann**

Germany

**Angela Lek**

United States of America

**Richard Lemmers**

Netherlands

**David Leslie**

United Kingdom

**Corina Lesseur**

France

**Siu-wai Leung**

Macao

**William Lewis**

United States of America

**Symen Ligthart**

Netherlands

**Charlotte Ling**

Sweden

**Alexander Link**

Germany

**Ranlu Liu**

United States of America

**Mo-Fang Liu**

China

**Adana A Llanos**

United States of America

**Geover Lobo**

India

**Colin Logie**

Netherlands

**Qianjin Lu**

China

**Xiangfeng Lu**

United States of America

**Amaia Lujambio**

United States of America

**Katie Lunnon**

United Kingdom

**Robert Lyle**

Norway

**Heidi Lyng**

Norway

**Donia Macartney-Coxson**

New Zealand

**Jennifer Maccani**

United States of America

**Deborah Mackay**

United Kingdom

**Luca Magnani**

United Kingdom

**Eamonn Maher**

United Kingdom

**N. Mahmud**

United States of America

**Mellissa Mann**

Canada

**Antonio Maraver**

France

**Juliana Maricato**

United States of America

**Riccardo Marioni**

United Kingdom

**Carmen Marsit**

United States of America

**John Martens**

Netherlands

**Daniel Martinez-Arguelles**

Canada

**David Martino**

Australia

**Athena Matakidou**

United Kingdom

**Giuseppe Matullo**

Italy

**Robert McKnight**

United States

**Allan Mcrae**

Australia

**Richard Meehan**

United Kingdom

**Gunther Meinlschmidt**

Switzerland

**L.J. Melchers**

Netherlands

**Bodo Melnik**

Germany

**Maite Mendioroz**

Spain

**Gerlinde Metz**

Canada

**Alexander Michels**

United States of America

**Daniel Miller**

United States of America

**Sheikh Mimi**

United Kingdom

**Svetlana Minakhina**

United States of America

**Raghavendra Mirmira**

United States of America

**Peter Molloy**

Australia

**Santiago Montes Moreno**

Spain

**Gudrun Moore**

United Kingdom

**Monika Morak**

Denmark

**Eva Morales**

Spain

**Valeria Motta**

Italy

**Sofia Movérare-Skrtic**

Sweden

**Anna Murray**

United Kingdom

**Adele Murrell**

United Kingdom

**Alessio Naccarati**

Italy

**Kari Nadeau**

United States of America

**Balint Nagy**

Hungary

**Kazuhiko Nakabayashi**

Japan

**Javad Nazarian**

United States of America

**Mihai Niculescu**

United States of America

**Emma Nilsson**

Sweden

**Jonas Nilsson**

Sweden

**Stephen Nimer**

United States of America

**Shuji Nomoto**

Japan

**Boris Novakovic**

Australia

**Andreas Nussler**

Germany

**Shuji Ogino**

United States of America

**Gabriel Oh**

Canada

**Jun Ohgane**

Japan

**Joyce Ohm**

United States of America

**Carla Oliveira**

Portugal

**Fabiola Olivieri**

Italy

**Miina Ollikainen**

Finland

**Francisco Ortega**

Spain

**Sasha Parets**

United States of America

**Paola Parrella**

Italy

**Marcelina Parrizas**

Spain

**Arpad V. Patai**

Hungary

**Veeresh K Patil**

United Kingdom

**Samir Patra**

India

**Sofia Pavanello**

Italy

**Kevin Pearson**

United States of America

**Mattia Pelizzola**

Italy

**Paivi Peltomäki**

Finland

**Paola Perego**

Italy

**Marcello Persico**

Italy

**Robert Philibert**

United States of America

**J. Richard Pilsner**

United States

**Hugo Pinheiro**

Portugal

**Carlos Pirola**

Argentina

**Flemming Pociot**

Denmark

**Dirk Prawitt**

Germany

**Julian Puppe**

Germany

**Yang Qin**

China

**Lyudmila Rachek**

United States of America

**Stacey Raimondi**

United States of America

**Emma Raitoharju**

Finland

**Agnieszka Rawłuszko-Wieczorek**

Poland

**Yasser Riazalhosseini**

Canada

**Rasmus Ribel-Madsen**

Denmark

**Christopher Ricketts**

United States of America

**Wendy Robinson**

Canada

**Ana Robles**

United States of America

**Euan Rodger**

New Zealand

**Silvia Rogatto**

Brazil

**Tina Rönn**

Sweden

**Christophe Rosty**

Australia

**Peter Rotwein**

United States of America

**Carlos rovira**

Sweden

**Joanne Ryan**

Australia

**Sonia Saad**

Australia

**Richard saffery**

Australia

**Gilles Salbert**

France

**Donato Santovito**

Italy

**Carmen Sapienza**

United States of America

**Shun Sato**

Japan

**Beat Schäfer**

Switzerland

**Leonard Schalkwyk**

United Kingdom

**Wolfgang Schulz**

Germany

**Stuart Scott**

United States of America

**Jurgen Serth**

Germany

**Karolina Setyowati**

Singapore

**Jing Shen**

United States of America

**Boris Arkadievich Shenderov**

Russia

**Rachna Shroff**

United States of America

**Roderick Slieker**

Netherlands

**Harold Snieder**

Netherlands

**Yuhu Song**

China

**Karina Dalsgaard Sørensen**

Denmark

**Régine Steegers-Theunissen**

Netherlands

**Renske Steenbergen**

Netherlands

**Olafur Stefansson**

Iceland

**Clare Stirzaker**

Australia

**Michael P Stone**

United States of America

**Susan R Sturgeon**

United States of America

**Isao Suetake**

Japan

**Hiroko Sugawara**

Japan

**Karsten Suhre**

France

**Saraswati Sukumar**

United States of America

**Deqiang Sun**

United States of America

**Yan Sun**

United States of America

**Cecilie Svanes**

Norway

**Atsushi Tajima**

Japan

**Rie Takahashi**

United States of America

**Rajeev Taliyan**

India

**Qihua Tan**

Denmark

**Satoshi Tanaka**

Japan

**Kristen Taylor**

United States of America

**Mathewos Tessema**

United States of America

**Vincent Thijs**

Belgium

**Stefania Tommasi**

Italy

**Marcio Alberto Torsoni**

Brazil

**Jorg Tost**

France

**Eric Toussirot**

France

**Fabiola Traina**

Brazil

**Philipp Tropberger**

United States of America

**Jiajie Tu**

China

**Rossella Tupler**

United States of America

**Gustavo Turecki**

Canada

**Bryan Turner**

United Kingdom

**Santiago Uribe-Lewis**

United Kingdom

**Toshikazu Ushijima**

Japan

**Valentina Vaira**

Italy

**Susan Van dijk**

Australia

**Hoang Van Tong**

Germany

**Donata Vercelli**

United States of America

**Mukesh Verma**

United States of America

**Sarah Voisin**

Sweden

**Paul Vrana**

United States of America

**Jun Wada**

Japan

**Graham Wallace**

United Kingdom

**Jin Wang**

China

**Jiucun Wang**

China

**Gaofeng Wang**

United States of America

**Ju Wang**

United States of America

**Xiaoling Wang**

United States of America

**Zhanwei Wang**

United States of America

**Robert Waterland**

United States of America

**Eugene Wee**

Australia

**Marco Weinberg**

South Africa

**Rosanna Weksberg**

Canada

**Antje Weseler**

Netherlands

**Gunnar Westin**

Sweden

**Stefan Wiemann**

Germany

**Stefan Willems**

Netherlands

**Ola Winqvist**

Sweden

**Bea Wisman**

Netherlands

**Justin Wong**

Australia

**Chun-Ming Wong**

Hong Kong

**KY Wong**

Hong Kong

**C Woodman**

United States of America

**Maria J. Worsham**

United States of America

**Wenji Yan**

China

**Rongxi Yang**

Germany

**Ivana Yang**

United States of America

**Xiaofei Yu**

United States of America

**Jian Yu**

China

**Youwei Yue**

China

**Nadia Zaffaroni**

Italy

**Adam Zawada**

Germany

**Amer Zeidan**

United States of America

**Zhi-Gang Zhang**

China

**Rongxin Zhang**

China

**Yuqi Zhao**

United States of America

**Wei-Guo Zhu**

China

**Inti Zlobec**

Switzerland

